# Comorbidities in polymyalgia rheumatica: a systematic review

**DOI:** 10.1186/s13075-018-1757-y

**Published:** 2018-11-20

**Authors:** Richard Partington, Toby Helliwell, Sara Muller, Alyshah Abdul Sultan, Christian Mallen

**Affiliations:** Arthritis Research UK Primary Care Centre, Primary Care Sciences, Keele University, Keele, ST5 5BG UK

**Keywords:** Polymyalgia rheumatica, Giant cell arteritis, Systematic review, Comorbidities, Multimorbidity, Epidemiology

## Abstract

**Background and aim:**

Comorbidities are known to exist in many rheumatological conditions. Polymyalgia rheumatica (PMR) is a common inflammatory rheumatological condition affecting older people which, prior to effective treatment, causes severe disability. Our understanding of associated comorbidities in PMR is based only on case reports or series and small cohort studies. The objective of this study is to review systematically the existing literature on the comorbidities associated with PMR.

**Methods:**

MEDLINE, EMBASE, PsycINFO and CINAHL databases were searched for original observational research from inception to November 2016. Papers containing the words ‘Polymyalgia Rheumatica’ OR ‘Giant Cell Arteritis’ OR the terms ‘PMR’ OR ‘GCA’ were included. Article titles were reviewed based on pre-defined criteria by two reviewers. Following selection for inclusion, studies were quality assessed using the Newcastle–Ottawa tool and data were extracted.

**Results:**

A total of 17,329 papers were reviewed and 41 were incorporated in this review, including three published after the search took place. Wide variations were found in study design, comorbidities reported and populations studied. Positive associations were found between PMR diagnosis and stroke, cardiovascular disease, peripheral arterial disease, diverticular disease and hypothyroidism. Two studies reported a positive association between PMR and overall malignancy rate. Seven studies reported an association between PMR and specific types of cancer, such as leukaemia, lymphoma, myeloproliferative disease and specified solid tumours, although nine studies found either no or negative association between cancer and PMR.

**Conclusion:**

Quantification of the prevalence of comorbidities in PMR is important to accurately plan service provision and enable identification of cases of PMR which may be more difficult to treat. This review highlights that research into comorbidities in PMR is, overall, methodologically inadequate and does not comprehensively cover all comorbidities. Future studies should consider a range of comorbidities in patients with a validated diagnosis of PMR in representative populations.

**Electronic supplementary material:**

The online version of this article (10.1186/s13075-018-1757-y) contains supplementary material, which is available to authorized users.

## Introduction

Polymyalgia rheumatica (PMR) is the most common inflammatory rheumatological condition affecting people over the age of 50 years [1]. Symptoms include muscle stiffness and pain, predominantly around the neck or shoulder and pelvic girdles [2], as well as a low-grade fever, depression, fatigue, anorexia and weight loss [3, 4]. Raised inflammatory markers (erythrocyte sedimentation rate (ESR) or C-reactive protein (CRP)) are a hallmark of this condition. PMR is usually treated with medium/low dose oral glucocorticoids (GCs) which are gradually reduced and stopped over several years [5].

Patients with common inflammatory rheumatological conditions, for example gout [6] and rheumatoid arthritis (RA) [7], are predisposed to developing cardiovascular disease (CVD). In patients with RA, this risk has been attributed to an increased prevalence of arterial atherosclerotic plaques [8, 9], the quantity of which are correlated with levels of systemic inflammation [10] and duration of rheumatological disease [11]. Patients with RA are also known to have a higher risk of lung diseases [12] and certain types of cancers, particularly haematological cancers [13]. PMR, like RA, is a rheumatological condition characterised by increased levels of inflammation, and therefore patients with PMR may have a similar predisposition to increased risks of certain conditions.

In order to diagnose PMR, guidelines endorsed by the American College of Rheumatology and the European League Against Rheumatism advise the exclusion of conditions which may cause similar symptoms [14]. These include core exclusion conditions (GCA, cancer and infections) as well as RA, fibromyalgia, hypothyroidism and drug-induced myalgia. The guidelines also suggest an evaluation of whether patients have comorbidities that put them at greater risk of side effects from GC treatment [14]. Quantifying the burden of comorbidities in this group of patients is therefore important.

The age group (typically over 50 years) most commonly affected by PMR frequently has more than one comorbidity. Aging is an important predictor of multimorbidity; a recent Scottish study found the number of adults with two or more chronic conditions increased from 30.4% between age 45 and 64 years, to 64.9% in those aged 65–84 years, to greater than 80% in those aged over 85 years [15]. This systematic review aims to summarise the available evidence of the comorbidity profile of people with PMR, and will be the first review to assess comprehensively the evidence for all comorbidities and whether there is evidence for multiple comorbidities existing together. If the evidence shows that patients with PMR commonly have multiple comorbidities then these may no longer be viewed as exclusion criteria precluding a diagnosis of PMR, potentially revealing the true burden of PMR to be higher than currently recognised.

## Methods

We conducted a systematic review and narrative synthesis of research literature. We searched medical bibliographic databases to identify articles containing data on any comorbidity either preceding or following a diagnosis of PMR.

### Data sources, searches and study selection

The search was conducted in MEDLINE, EMBASE, PsyciINFO and CINAHL from their inception until the date of search in November 2016. Additional articles were found by examining reference lists of included studies and an updated search was run in June 2018 which led to the inclusion of a further study. The exploded MeSH terms ‘polymyalgia rheumatica’ and ‘giant cell arteritis’ were used in combination with text word searches for the same as well as for ‘PMR’ and ‘Giant Cell Arteritis’ (GCA). GCA is a vasculitis which very commonly co-occurs with PMR; around 10–30% of patients with PMR develop GCA during the course of their illness [16, 17]. Given this overlap in conditions, GCA was included to increase the likelihood of ensuring that all studies in which PMR comorbidities were considered were included in the review. PRISMA guidelines were followed throughout the review process [18].

All article titles identified were screened by a single reviewer (RP) against the inclusion and exclusion criteria. A random sample of 100 of these titles was reviewed by a second reviewer (TH) and agreement between decisions was assessed using adjusted κ calculation [19]. All selected abstracts were then assessed by two reviewers (RP and TH). Any citation thought to be eligible by either reviewer was carried forward to full text review. Reasons for exclusion were recorded. Finally, the remaining full texts were reviewed by the same two authors and a list of papers to be included in the narrative synthesis was created.

### Inclusion and exclusion criteria

The inclusion criteria for this review included: a sample of patients with PMR and at least one comorbidity; and the study design must be either cross-sectional, case–control or a prospective or retrospective cohort study. Exclusion criteria were: patients under the age of 40 years; randomised control trials (RCTs); and review articles or conference abstracts. PMR is a disease of older adults. In order to make a diagnosis of PMR, clinical guidelines suggest patients must be aged over 50 years [20], therefore patients under 40 years old are likely to represent misdiagnosis. RCTs were excluded as we wished to look at representative samples of patients with PMR drawn from real-world, observational data. Review articles and conference abstracts were not included to ensure all articles were peer reviewed and fully referenced. In order to ensure that all conditions represented true comorbidities, rather than secondary complications of GC treatment in PMR, we excluded trials which reported only complications of GC therapy [3, 21–27].

There were no date or language restrictions although all included studies were in English. Potentially relevant studies that contained data on GCA were included until full text review due to the overlap between PMR and GCA. If, at that point, the paper only contained data about GCA, it was excluded. The reference lists of other systematic reviews that had assessed individual comorbidities related to PMR were also reviewed to reduce the chance of missing relevant studies.

### Quality assessment

Both reviewers, using the Newcastle–Ottawa Scale for case–control and/or cohort studies [28], evaluated the quality of studies. This scale was chosen as it is endorsed for use in systematic reviews of non-randomised trials by the Cochrane Collaboration [29].

### Data extraction

A standardised form was developed and used by both reviewers independently to ensure the accuracy of data extraction (Additional file 1). The primary outcome of interest was the total number of patients with PMR who developed a comorbidity of interest compared to controls (without PMR). Other data extracted included clinical criteria used to diagnose PMR, study design, comorbidity under investigation and its temporal relationship to PMR. Meta-data from each study, such as lead author name, publication year, sex, age, country and healthcare setting, were also extracted. Comorbidities were categorised into four groups: malignant disease, particularly haematological malignancies; vascular disease, including coronary, cerebroarterial and peripheral arterial disease; mortality; and other comorbidities (e.g. endocrine, psychiatric and neurological diseases).

### Data analysis

Using the total number of patients with PMR and, if present, their controls we attempted to aggregate data to obtain pooled estimates of prevalence of comorbidities and odds ratios to quantify the strength of any apparent association.

## Results

### Search results

A total of 27,698 articles were identified in this search with a further seven identified following review of references of other articles. Of this total, 10,376 were removed due to duplications, leaving 17,329 unique articles. Following application of inclusion and exclusion criteria during screening of titles, 17,042 further citations were excluded. Of the random selection of 100 of these articles which were reviewed by a second author, agreement between authors was excellent (κ = 0.86).

**Fig. 1:**
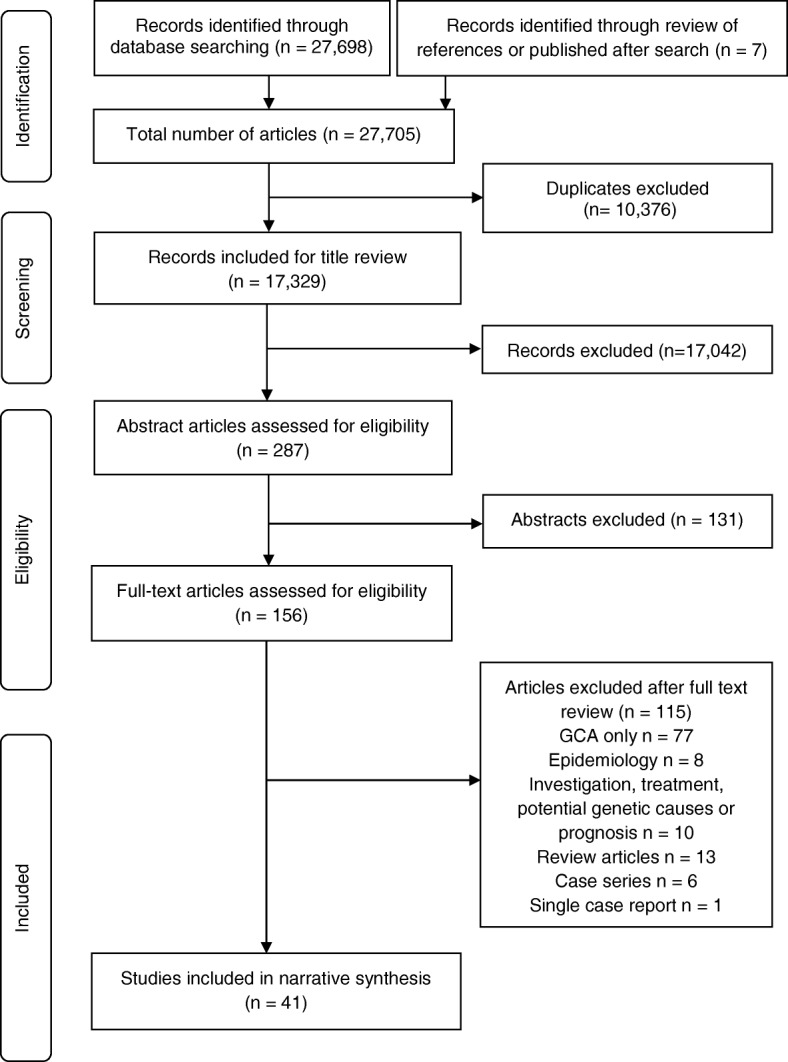
Flowchart of study inclusion, adopted from Preferred Reporting Items for Systematic Reviews and Meta-Analyses (PRISMA) guidelines [18]. GCA giant cell arteritis

The abstracts of 287 studies were assessed for eligibility, and 131 were excluded at this stage. The full texts of articles were reviewed and 41 were retained for data extraction [30–70]. This process, which followed PRISMA guidelines, including reasons for exclusion of studies [18], is illustrated in Fig. 1.

### Articles included in the review

Of the 41 included studies, 32 were cohort studies [30–61] and nine were case–control studies [62–70]. Eighteen of the cohort studies did not use a formal comparator group. Of the 14 cohort studies with controls, six were based on national datasets, whereas eight were based either on local datasets or on patients presenting to clinics at the same hospital. PMR cases were defined from medical records in 16 studies, national registries in 19 studies and national databases in the remaining six studies. Co-existent GCA cases were formally excluded in six studies and included, or not explicitly excluded, in 35 studies. All but one study was from Europe (predominantly Scandinavia) or the United States. Included studies are tabulated in Additional file 2.

### PMR and cancer

Seven studies, reporting 12 outcome measures, have assessed the risk of cancer diagnosis prior to PMR onset (Table 1). All of these studies excluded PMR diagnoses made in the year prior to diagnosis of cancer, to reduce the risk of reverse causality (i.e. cancer causing PMR or PMR symptoms). Of these, the rate of haematogenous cancers was significantly higher among patients with PMR in five cases, while the other seven were non-significantly different.

**Table 1:** Cancer prior to diagnosis with PMR

Retrospective case–control study								
Study	Diagnosis	Cases (*n*)	PMR cases (*n*)	Controls (*n*)	PMR controls (*n*)	Odds ratio (95% CI)	Case rate (%)	Control rate (%)
Anderson et al. [64]	Lymphoid malignancies	33,721	344	122,531	1244	0.9 (0.8–1.0)	1.02	1.02
Anderson et al. [63]	Myeloid malignancy	9998	125	160,086	1288	1.7 (1.4–2.1)	1.25	0.80
	Myelodysplastic malignancy	3758	55	42,886	518	1.5 (1.1–2)	1.46	1.21
Anderson et al. [67]	HCL	418	9	160,086	2721	1.5 (0.5–3.9)	2.15	1.70
Askling et al. [62]	All lymphoma	42,676	114	78,487	250	0.8 (0.7–1.0)	0.27	0.32
	NHL	28,355	88	52,164	187	0.9 (0.7–1.1)	0.31	0.36
	HL	4037	3	7394	15	0.4 (0.1–1.3)	0.07	0.2
	CLL	10,555	24	19,391	52	0.8 (0.5–1.4)	0.23	0.27
Kristinsson et al. [66]	Any MPN	11,039	46	43,550	104	1.7 (1.2–2.5)	0.42	0.24
Lanoy and Engels [65]	Cutaneous NHL	2652	19	178,452	1731	0.7 (0.5–1.1)	0.72	0.97
Lindqvist et al. [68]	MM	19,112	56	75,408	116	1.9 (1.4–2.6)	0.29	0.15
	MGUS	5403	58	21,209	79	2.9 (2.1–4.1)	1.07	0.37

*PMR* polymyalgia rheumatica, *HCL* hairy cell leukaemia, *NHL* non-Hodgkin’s lymphoma, *HL* Hodgkin’s lymphoma, *CLL* chronic lymphocytic leukaemia, *MM* multiple myeloma, *MPN* myeloproliferative neoplasm, *MGUS* monoclonal gammopathy of undetermined significance

Six studies reported prospective rates of any cancer diagnosis after diagnosis with PMR (Table 2); two showed an increase in the proportion of people with PMR who developed cancer compared to controls, two were equivocal and the remaining two found the opposite.

**Table 2:** Cancer following diagnosis with PMR

Prospective cohort with combined cancer cases						
	PMR patients			Control patients		
Study	PMR cases (*n*)	Cancer cases (*n*)	Proportion (%)	Controls (*n*)	Cancer cases (*n*)	Proportion (%)
Muller et al. [57]	2877	667	23.18	9942	1938	19.49
Bellan et al. [61]	100	24	24.00	702	41	5.84
Ji et al. [45]	35,918	3941	10.97	–	–	–
Myklebust et al. [37]	366	34	9.29	1324	143	10.80
Haga et al. [32]	91	10	10.99	794	131	16.50
Pfeifer et al. [59]	359	66	18.38	357	62	17.37
Total^a^	39,711	4742	21.12	13,119	2315	17.65

*PMR* polymyalgia rheumatica

^a^Ji et al. [45] not included in calculation as no control group

In six prospective cohort studies the risk of 17 types of cancer following diagnosis with PMR was considered. Two studies showed an increase in the risk of Hodgkin’s lymphoma [55] and non-Hodgkin’s lymphoma [56]. Of the remaining four studies, three reported no difference in the rates of female cancers [51], upper gastrointestinal cancers [47] or myeloma [54]. The final study reported no difference in mortality following diagnosis with gastrointestinal cancers [50] among people with or without PMR.

### PMR and vascular disease

A number of studies (*n* = 8) have assessed a variety of different vascular diseases in patients with PMR (Table 3). Fifteen outcome measures were reported, although only seven gave comparable figures for patients without PMR. In each study with a comparator group the proportion of people with PMR who developed vascular disease was higher compared to controls.

**Table 3:** Vascular disease and PMR

	PMR patients			Control patients		
Study	PMR cases (*n*)	Comorbid condition (*n*)	Proportion (%)	Controls (*n*)	Comorbid condition (*n*)	Proportion (%)
Stroke						
Kang et al. [46]	781	113	14.47	3905	273	6.99
Zoller et al. [48]	16,496	1981	12.01			
Kremers et al. [42]	276	58	21.01			
Hancock et al. [58]	3249	397	12.22	12,735	556	4.37
Myocardial infarction						
Kremers et al. [42]	276	47	17.03			
Hancock et al. [58]	3249	460	14.16	12,735	575	4.52
Zoller et al. [48]	21,351	5669	26.55			
Heart failure						
Kremers et al. [42]	276	68	24.64			
Peripheral vascular disease						
Kremers et al. [42]	276	35	12.68			
Hancock et al. [58]	3249	140	4.31	12,735	151	1.19
Warrington et al. [43]	353	38	10.76	705	28	3.97
Combined						
Kremers et al. [42]	276	208	75.36			
Hancock et al. [58]	3249	918	28.25	12,735	1150	9.03
Pujades-Rodriguez et al. [60]	9776	2272	23.24	105,504	21,559	20.43
Bengtsson and Malmvall [30]	73	16	21.92			

*PMR* polymyalgia rheumatica

### PMR and mortality

Few studies have assessed the association between PMR and mortality (*n* = 4). Three studies reported reduced mortality among patients diagnosed with PMR [35, 38, 39] while one study found an increase, but this study did not differentiate between patients with PMR and GCA [36].

### PMR and other comorbidities

An association between thyroid disease and PMR is unproven. Bowness et al. [31] found an increase in the risk of hypothyroid disease (RR 3.2 (95% confidence interval 1.71, 5.91)), but Juchet et al. [33] did not. One recent case–control study found a significantly increased rate of diverticular disease prior to a diagnosis with PMR (OR 4.06 (95% CI 1.76–9.35)) [70].

No evidence has been found to associate PMR with psychiatric comorbidities, including schizophrenia [44, 69] and bipolar disease [44]. Li et al. [49] found a potential association between PMR and Parkinson’s disease (SIR 1.25 (95% CI 1.01, 1.53)). Hemminki et al. [53] also reported an association between PMR and hospitalisation due to obesity (SIR 1.65 (95% CI 1.22, 2.19)).

A small number of studies from the United States (*n* = 2) [40, 59] looked at wide ranges of different comorbidities but their sample size was insufficient to find significant associations for the majority of the comorbidities.

### Quality assessment

All of the articles in this study used medical records or nationwide registries (based on medical records) to corroborate diagnosis of PMR and the comorbidity; therefore, they were awarded three or four stars for cohort or case selection using the Newcastle–Ottawa criteria. All studies also achieved at least two stars for outcome measurement. However, many of the studies failed to recruit a comparator group, instead using the population as a reference, and therefore comparability scores were low (Additional file 2).

Many of the cohort studies identified failed to include comparison groups (*n* = 18), instead using indirect standardisation to calculate incidence or mortality ratios. The lack of comparison groups limits the generalisability of many of the studies. Further to this, almost half of the studies (*n* = 19) sourced their sample of patients with PMR based on hospital discharge data. This may be an appropriate approach for some autoimmune conditions, but the majority of patients with PMR are managed in primary care settings [71, 72].

Aggregation of data to calculate pooled odds and hazard ratios was attempted for vascular disease and cancer diagnoses; however, high levels of heterogeneity were found between the studies (88–100%) and therefore this was not reported.

Many of the studies limited themselves to a small number of comorbid conditions, thus not allowing a picture of the overall health of patients with PMR to develop. Two studies [34, 40] did attempt to look at a range of comorbidities but they were underpowered.

## Discussion

### Statement of principle findings

This review found some evidence of an association between PMR and vascular disease, and possibly cancer, particularly in the first 6 months following diagnosis. However, the evidence for this is not robust.

The concentration of the apparent association between PMR and cancer in the first 6 months following diagnosis suggests the possibility of an element of misdiagnosis. This could occur as some of the features of PMR (myalgia, fatigue, weight loss, raised inflammatory markers) are also non-specific early features of some cancers. Furthermore, as time passed, the rate of diagnosis of cancer was found to drop down to the background population rate.

Regarding specific types of cancer, some studies have proposed there could be associations between PMR and haematological cancers. This includes Hodgkin’s and non-Hodgkin’s lymphoma [56], myeloma [68] and other myeloid malignancies [63, 66]. An increase in the risk of lymphoma has been observed with RA, which has been postulated to be due to higher accumulated inflammatory activity in RA [73]; a similar mechanism may lie behind the apparent increase in patients with PMR.

The overall trend of results suggests that PMR may be associated with an increased risk of the development of vascular disease. Knowing that both PMR and RA are inflammatory conditions, there is biological plausibility that PMR and vascular disease could be associated. However, the two largest studies, both based on population data from the UK, reported conflicting results: Hancock et al. [58] stated that PMR was significantly associated with vascular disease, while Pujades-Rodriguez et al. [60] reported a reduction in the risk (incidence rate ratio 0.88 (95% CI 0.83–, 0.94)). However, in the latter study when only patients with PMR were included, there was a slight increase in the proportion of patients with the condition who went on to have a vascular event compared to controls (23.24% compared to 20.43%).

These two studies employed similar approaches selecting with PMR from linked UK databases. However, a number of differences existed between the studies, including the age of participants (> 50 years only for Hancock et al. [58] and > 18 years for Pujades-Rodriguez et al. [60]), average years of follow up (7.8 and 3.13 years respectively) as well as the total number of patients found with PMR (3249 compared to 11,320 patients). Potentially, the variation in risk of vascular events between these studies could be explained by the differences in follow up or the age distribution of the study population.

Another reason for inconsistent evidence of an increase in vascular risk for patients with PMR may be the modulating effect that GCs have upon levels on inflammation. If the risk of vascular disease correlates with the presence of inflammation in the body, GC therapy would reduce this, which may then also reduce vascular risk. The study by Kremers et al. [42] appears to bear this out. In this study, the risk of vascular events was lower in patients with PMR who were treated with GCs compared to those who were not. Further to this, Hancock et al. [58] reported that the excess vascular risk in PMR reduced over time; this could reflect declining levels of inflammation.

Overall, although some studies dissent from this view, it appears that a diagnosis of PMR increases an individual’s risk of vascular disease. However, further research in this area is needed to add clarity.

Conversely, it appears that a diagnosis with PMR is associated with a reduction in mortality. This was demonstrated in three out of the four studies that reported it as an independent outcome. A possible explanation for this could again be surveillance bias. Patients with chronic illness (and especially PMR where regular assessment, follow up and monitoring are advised) are more likely to be under active follow up for their condition and any developing morbidity, particularly if related to well-recognised adverse effects of treatment, is likely to be identified and managed earlier.

There is a small amount of evidence that patients with PMR may be more likely to develop hypothyroidism [31] and Parkinson’s disease [49]. PMR and hypothyroidism both preferentially affect females, and therefore a similar autoimmune pathway may be present in both conditions. However, as has been pointed out, PMR does not share all of the characteristics of traditional autoimmune conditions, for example it lacks specific autoantibodies [74]. Furthermore, it seems that PMR is not associated as strongly with other autoimmune conditions as would be expected if it was a pure autoimmune disease. Parkinson’s disease is a condition which predominantly affects older people, as does PMR, and therefore this association may just be a result of clustering of diagnoses in an older population.

### Strengths and weaknesses

The main strength of the study was its deliberately broad scope and aim to include any study in which the risk of any comorbidity either before or after diagnosis with PMR was to be reviewed. Following the initial broad search, articles from the ‘grey literature’ were excluded and only articles fully published in peer-reviewed journals were included.

The potential limitations in this study arose not due to the protocol but rather because the majority of studies were of relatively poor quality. These risks included selection bias, surveillance bias and a lack of adequate control groups.

Selection bias within the included studies is a possibility in this review, as the majority of studies drew PMR cases from secondary care, either from hospital discharge data or from rheumatology outpatient clinics. Current UK guidelines suggest only referring atypical cases, cases of diagnostic uncertainty or treatment predicaments [5, 14], meaning that in the UK 71–84% of patients with PMR are treated in the community by primary care physicians [71, 72]. Therefore, the patient population in these studies may not accurately reflect the majority of those who are diagnosed with PMR. This may have artificially inflated the apparent differences in development of comorbidities between patients with and without PMR.

Another limitation is the risk of surveillance bias as discussed earlier around the apparent reduction in mortality [75]. Some case–control studies attempted to deal with this by excluding comorbid disease found in the year prior to diagnosis [63, 64, 67, 68], while in two observational cohort studies [58, 60], controls were selected that had contacted a primary care service in the year the index cases were diagnosed. Finally, many of these studies assessed multiple variables, often > 30 different autoimmune conditions, increasing the likelihood of a chance finding (type II error).

Furthermore, we also noted that the range of comorbidities reported in the literature was more limited than we expected; for example, there were no studies which explicitly examined the risks of important and common conditions such as diabetes mellitus and asthma or other chronic respiratory conditions.

A further potential bias is the effect of GC therapy and the impact of this on the risks of comorbidities. As GC is the only widely accepted treatment for PMR, we could not exclude studies where patients were treated with GC. To reduce the impact of potential bias from GC treatment, we excluded studies in which direct complications of GC therapy were assessed. However, as discussed previously in relation to vascular risk, GC therapy is inevitable in PMR and therefore we could not completely mitigate this effect.

## Conclusion

This review has found the overall standard of evidence regarding the association of comorbidities with PMR to be weak. There may be an increased risk of vascular disease and possibly cancer in patients with PMR. Weaker quality evidence also suggests that patients with PMR have a reduced mortality rate. Currently, there is little evidence around the wider health of patients with PMR either at the time of diagnosis or in the period following.

This lack of firm evidence around which comorbidities exist alongside or are potentially associated with PMR presents a problem for the pragmatic clinician. Current clinical guidelines suggest that in order to diagnose PMR, a large number of other conditions which may mimic the symptoms of PMR should be excluded. This list includes, but is not limited to, rheumatoid arthritis and endocrine, infective and neoplastic conditions [14]. However, comorbidities are very common in the age group affected by PMR [76], and therefore the coexistence of one of these comorbidities with PMR should not necessarily prevent or invalidate the diagnosis of PMR.

The uncertainty around the general health of patients with PMR and comorbidities that may coexist with it presents a challenge for healthcare practitioners who deal most with this condition, be they from primary or secondary care. A rigorous diagnostic and follow-up process is crucial to ensure this uncertainty does not translate into misdiagnosis. In the future, it is important to confirm whether, and if so to what extent, a diagnosis of PMR imparts an excess risk of vascular disease or cancer.

Further research in the form of large observational studies, based in primary care, of the health of patients with PMR, including the prevalence of comorbidities before and after diagnosis, would allow clinicians to better monitor for these outcomes.

## Additional files

Additional file 1: Data collection form (DOCX 18 kb)

Additional file 2: Details of all included studies (DOCX 72 kb)

## Abbreviations

CRP: C-reactive protein
CVD: Cardiovascular disease
ESR: Erythrocyte sedimentation rate
GC: Glucocorticoid
GCA: Giant cell arteritis
PMR: Polymyalgia rheumatica
RA: Rheumatoid arthritis
RCT: Randomised control trial
