# Supplementary material for: Comorbidities in polymyalgia rheumatica: a systematic review
Source: Arthritis Res Ther. 2018 Nov 20;20:258. doi: 10.1186/s13075-018-1757-y (PMC6247740; doi:10.1186/s13075-018-1757-y)
Supplement: Supplementary file 1 — Data collection form (DOCX 18 kb) [file 13075_2018_1757_MOESM1_ESM.docx]

Additional file 1: Data collection form

| **Study ID** |  | |
| --- | --- | --- |
| **Author** |  | |
| **Title** |  | |
| **Journal** |  | |
| **Year** |  | |
| **Volume** |  | |
| **Issue** |  | |
| **Page numbers** |  | |
| **Language of publication** |  | |
| **Continent where data collection occurred** |  | |
| **Population** | PMR: | Controls: |
| **Age** | PMR: | Controls |
| **Sex** | PMR: | Controls |

| **PMR Clinical Criteria Used** |  | **Study design** |  |
| --- | --- | --- | --- |
| Chuang |  | Cohort |  |
| Bird |  | Case control |  |
| Jones |  | Cross sectional |  |
| Nobunaga |  | Population |  |
| Healey |  | Systematic Review |  |
| Hunder |  |  |  |
| EULAR-ACR |  |  |  |
| Not referenced |  |  |  |

| **Name of comorbidity(s)** | **Retrospective** | **Prospective** |
| --- | --- | --- |
|  |  |  |
|  |  |  |
|  |  |  |
|  |  |  |
|  |  |  |

|  | N |  | N |
| --- | --- | --- | --- |
| Cases |  | Controls |  |
| Comorbid condition |  | Comorbid condition |  |
| Proportion affected / Case rate |  | Proportion affected / Case rate |  |
| Person years follow up |  | Person years follow up |  |
| Incidence rate / Odds |  | Incidence rate / Odds |  |
| Incidence rate or Odds ratio |  |  |  |
| Effect Measures from paper |  |  |  |
