# Supplementary material for: Comorbidities in polymyalgia rheumatica: a systematic review
Source: Arthritis Res Ther. 2018 Nov 20;20:258. doi: 10.1186/s13075-018-1757-y (PMC6247740; doi:10.1186/s13075-018-1757-y)
Supplement: Supplementary file 2 — Details of all included studies (DOCX 72 kb) [file 13075_2018_1757_MOESM2_ESM.docx]

Additional file 2: All included studies

| Author | Study Location | Year | Study Type | Comorbidity(s) assessed | Temporal Relationship of Comorbidity | Quality Assessment | | |
| --- | --- | --- | --- | --- | --- | --- | --- | --- |
|  |  |  |  |  |  | Selection | Comparability | Outcome |
| Bengtsson^30^ | Sweden | 1981 | Cohort | Vascular events | Prospective | 4 | 0 | 2 |
| Bowness^31^ | United Kingdom | 1991 | Cohort | Thyroid disease | Prospective | 4 | 0 | 2 |
| Juchet^33^ | France | 1993 | Cohort | Thyroid disease | Prospective | 3 | 0 | 2 |
| Haga^32^ | Norway | 1993 | Cohort | Cancer | Prospective | 4 | 1 | 2 |
| Schaufelberger ^34^ | Sweden | 1995 | Cohort | Mortality | Prospective | 4 | 0 | 2 |
| Gran ^35^ | Norway | 2001 | Cohort | Mortality | Prospective | 4 | 1 | 3 |
| Doran ^38^ | United States | 2002 | Cohort | Mortality | Prospective | 4 | 0 | 2 |
| Uddhamar ^36^ | Sweden | 2002 | Cohort | Mortality | Prospective | 3 | 0 | 3 |
| Myklebust ^37^ | Norway | 2002 | Cohort | All cancer | Both | 4 | 2 | 3 |
| Myklebust ^39^ | Norway | 2003 | Cohort | Mortality | Prospective | 4 | 1 | 3 |
| Askling ^62^ | Sweden | 2005 | Case Control | Lymphoma | Retrospective | 3 | 2 | 3 |
| Kremers ^40^ | United States | 2005 | Cohort | Various | Prospective | 4 | 2 | 3 |
| Eaton ^41^ | Denmark | 2007 | Cohort | Autoimmune diseases | Cross-sectional | 4 | 0 | 2 |
| Kremers ^42^ | United States | 2007 | Cohort | Vascular events | Prospective | 3 | 0 | 2 |
| Warrington ^43^ | United States | 2009 | Cohort | Peripheral Vascular Disease | Prospective | 4 | 2 | 2 |
| Anderson ^64^ | United States | 2009 | Case Control | Lymphomas | Retrospective | 3 | 2 | 3 |
| Anderson ^63^ | United States | 2009 | Case Control | Myeloid malignancies | Retrospective | 3 | 2 | 3 |
| Anderson ^67^ | United States | 2010 | Case Control | Leukaemia | Retrospective | 3 | 2 | 3 |
| Ji ^45^ | Sweden | 2010 | Cohort | Malignancy | Prospective | 3 | 1 | 3 |
| Kristinsson ^66^ | Sweden | 2010 | Case control | Myeloproliferative neoplasms | Retrospective | 3 | 2 | 3 |
| Lanoy ^65^ | United States | 2010 | Case Control | Skin Cancers | Retrospective | 3 | 2 | 3 |
| Eaton ^44^ | Denmark | 2006 | Cohort | Psychiatric | Retrospective | 4 | 0 | 3 |
| Kang ^46^ | Taiwan | 2011 | Cohort | Stroke | Prospective | 4 | 2 | 2 |
| Lindqvist ^68^ | Sweden | 2011 | Case Control | Plasma cell cancers | Retrospective | 3 | 2 | 3 |
| Zoller ^48^ | Sweden | 2012 | Cohort | Stroke | Prospective | 4 | 0 | 3 |
| Zoller ^52^ | Sweden | 2012 | Cohort | Vascular events | Prospective | 4 | 0 | 2 |
| Hemminki ^47^ | Sweden | 2012 | Cohort | Digestive tract cancer | Prospective | 4 | 0 | 2 |
| Hemminki ^50^ | Sweden | 2012 | Cohort | Digestive tract cancer | Prospective | 4 | 0 | 2 |
| Hemminki ^51^ | Sweden | 2012 | Cohort | Female cancers | Prospective | 4 | 0 | 2 |
| Hemminki ^54^ | Sweden | 2012 | Cohort | Myeloma | Prospective | 4 | 0 | 2 |
| Chen ^69^ | Taiwan | 2012 | Case control | Schizophrenia | Retrospective | 3 | 2 | 3 |
| Li ^49^ | Sweden | 2012 | Cohort | Parkinson's disease | Prospective | 4 | 0 | 2 |
| Hemminki ^53^ | Sweden | 2012 | Cohort | Obesity (hospitalisation) | Prospective | 4 | 0 | 2 |
| Muller ^57^ | United Kingdom | 2013 | Cohort | All cancer | Prospective | 4 | 2 | 3 |
| Hancock ^58^ | United Kingdom | 2014 | Cohort | Vascular events | Prospective | 4 | 2 | 2 |
| Fallah ^56^ | Sweden | 2014 | Cohort | Non Hodgkins Lymphoma | Prospective | 4 | 0 | 2 |
| Fallah ^55^ | Sweden | 2014 | Cohort | Hodgkin lymphoma | Prospective | 4 | 0 | 2 |
| Pfeifer ^59^ | United States | 2015 | Cohort | All cancers | Both | 3 | 2 | 3 |
| Pujades-Rodriguez ^60^ | United Kingdom | 2016 | Cohort | Vascular events | Prospective | 4 | 2 | 2 |
| Bellan ^61^ | Italy | 2017 | Cohort | Cancer | Prospective | 3 | 0 | 3 |
| Scrivo^70^ | Italy | 2018 | Case control | Diverticular disease | Retrospective | 3 | 2 | 3 |
